# It is time to move: linking flight and foraging behaviour in a diving bird

**DOI:** 10.1098/rsbl.2007.0088

**Published:** 2007-05-15

**Authors:** David Pelletier, Magella Guillemette, Jean-Marc Grandbois, Patrick J Butler

**Affiliations:** 1 Département de biologie, Université du Québec à RimouskiRimouski, Québec, Canada G5L 3A1; 2 Centre for Ornithology, School of Biosciences, The University of BirminghamEdgbaston, Birmingham B15 2TT, UK

**Keywords:** common eider, data logger, diving behaviour, flying behaviour, free-living birds, heart rate

## Abstract

Although the adaptive value of flight may seem obvious, it is the most difficult behaviour of birds to monitor. Here, we describe a technique to quantify the frequency and the duration of flights over several months by implanting a data logger that records heart rate (*f*_H_), hydrostatic pressure (diving depth) and the body angle of a large sea duck species, the common eider (*Somateria mollissima*). According to the mean *f*_H_ recorded during flight and the parameters recorded to identify the *f*_H_ flight signature, we were able to identify all flights performed by 13 individuals during eight months. We cumulated local flight time (outside migrations) and found that activity occurs primarily during dawn and morning and that flying activities are strongly related to diving activities (Pearson's *r*=0.88, permutation test *p*<0.001). This relationship was interpreted as a consequence of living in a dynamic environment where sea currents move the ducks away from the food patches. We believe that the technique described here will open new avenues of investigation in the adaptive value of flight.

## 1. Introduction

The main functions of flight are to increase foraging success, reduce the probability of predation and facilitate long-distance movements to escape adverse conditions (Pomeroy 1990). However, to link flight to one of these advantages is difficult owing to technical and logistical difficulties. Radar and satellite telemetry have been used to analyse the orientation and duration of long migrating flights (Casement 1966; Butler *et al.* 1998; Hays *et al.* 2001), but none provided detailed information about flights of shorter duration. To quantify short local flights, researchers have used visual observations (Walsberg 1983) and telemetry (Ackerman *et al.* 2006), but these methods are probably biased for most species and ineffective to monitor marine birds.

For almost four decades, the fact that heart rate increases to high levels during forward flapping flight has been documented by physiologists studying both short (Lord *et al.* 1962; Kanwisher *et al*. 1978; Bevan *et al.* 1997) and migration flights (Butler *et al.* 1998). However, the heart rate (*f*_H_) signature of flight (shape of trace in relation to time) has never been quantified and used as a means of identifying and compiling all flights performed by a bird species.

We studied the flying and diving behaviour of common eider (*Somateria mollissima*), a large sea duck species that spend most of its time (11 months) at sea. We describe the characteristics of the flight signature and present data of observations during the post-hatching period in order to discriminate *f*_H_ reached during flight from that recorded during other behaviours. We show the potential of this new technique by analysing the occurrence of flight behaviour in relation to time of the day and foraging behaviour. We tested the hypothesis that flight behaviour in this species is mostly used as a way to improve foraging opportunities.

## 2. Material and methods

In 2003 and 2004, 20 and 10 female eiders, respectively, were equipped with data loggers (DLs, 36×28×11 mm; 21 g, i.e. less than 1.2% of body mass, Woakes *et al.* 1995). The DLs recorded heart rate (0.5 Hz), pressure (dive depth, 0.5 Hz) and body angle (1 Hz, those used in 2004 only). The body angle switch turned on due to acceleration and speed or when the DL is at an angle of +45 or −45°. Using a before–after design with a control group, it was impossible to detect any negative impact on laying date, clutch size and hatching success for individuals carrying a DL for one full year (Guillemette *et al.* 2002). Furthermore, the DLs used in this study were implanted in the body cavity of the experimental bird, with no external protrusions, thus conserving aerodynamic and hydrodynamic properties of the study animals (Guillemette *et al.* 2002).

Only females implanted in 2003 were included in the analysis of flight time. Eighteen females returned to the colony (90%) for laying, 17 of which were recaptured 1 year later and their DLs were removed. Only 13 loggers with their memory full or almost full (190–220 days) were kept for flight analysis, data spanning from May 2003 until the middle of December 2003. We kept only local flights to test our hypothesis (99% of all flights) that flight behaviour was mostly linked to foraging behaviour and demand.

We compared *f*_H_ recorded during flight to *f*_H_ recorded during different activities by conducting focal observations (only one animal at a time) on 10 (2003) and 4 (2004) experimental females during the post-hatching period (total of 118 h of observation). Behaviours were assigned to one of the 11 categories described in the electronic supplementary material. By using the signature of recorded flight activity performed during the post-hatching period, we found the set of parameters (figure 1) giving the best description of the shape of trace in relation to time, and they were programmed in a purpose-designed software for the extraction of all flights performed by each female. The set of parameters was then validated by visual analysis of traces in all recording days in each female to ensure that the parameters selected only the *f*_H_ signatures related to flight.

Owing to the extensive recording period, the duration of daylight varied along the sampling period. Thus, we had to adjust take-off time of each flight to determine the percentage of flights performed according to sunrise and sunset by using the equation: *adjusted time*=(*t*_0_−*H*_sunrise_)/(*H*_sunset_−*H*_sunrise_), where *t*_0_ is the take-off time, *H*_sunrise_ sunrise time and *H*_sunset_ sunset time (dawn is between −0.2 and 0.0, 0.0 is sunrise, 0.5 is midday, 1.0 is sunset, dusk is between 1.0 and 1.2, and outside these boundaries it is night).

## 3. Results

Parameters found to identify *f*_H_ flight signature during forward flapping flight were defined using focal observations and are summarized in figure 1. The almost instantaneous increase in *f*_H_ during take-off and the large decrease during landing are quantified with increasing and decreasing slopes equal to or above 10 beats per minute per second (in absolute values). The take-off ascending slope exceeded 250 beats per minute and the *f*_H_ plateau reached after take-off and recorded during forward flapping flight exceeded a threshold of 300 beats per minute. To eliminate some *f*_H_ events related to diving bouts or bathing behaviour with head submersion that are very similar to flights (Stephenson *et al.* 1986), we removed all *f*_H_ events with unstable hydrostatic pressure (standard deviation of diving depth equal to or above 0.1 m). The body angle switch turned on while birds were taking off from land, stayed on during the majority of plateau phase duration and turned off during the landing. We used these binary data to confirm the other parameters' thresholds. With this set of parameters, we could identify flights longer than 18 s with a plateau phase longer than 8 s.

Local flights for each female were performed primarily between sunrise and sunset (morning=39±2% (s.e.m.) and afternoon=31±6%; figure 2). However, a substantial number of flights were also performed during the night (14±6%), at dawn (12±5%) and dusk (5±2%). Local flights were of higher frequency mainly 55 min either side of sunrise (20±4%), declined steadily throughout the day and experienced a rapid decline at dusk. Females were flying on average 9.6±2.4 min d^−1^ outside migration periods, i.e. only 0.7% of the day.

Diving activities were performed more during the day (morning=42±5% and afternoon=40±6%) than during other periods (night=10±6%, dusk=5±2% and dawn=3±3%). As a result, the average flight and dive frequencies were highly correlated (Pearson's *r*=0.88, permutation test *p*<0.001; figure 2). The diving frequency reached its maximum value approximately 2 h after sunrise.

## 4. Discussion

In the present study, we provide, to our knowledge, the first identification of all flights and foraging events in a wild bird. It is easy to distinguish flights (duration longer than 18 s) from other activities using *f*_H_ data recorded by a DL in a bird performing forward flapping flight. The only activity that could be similar to flight is bathing behaviour, during which the bird is flapping its wings continuously and rapidly over the water and frequently submerging its head underwater. However, bathing behaviour, in spite of high mean *f*_H_, shows distinctive traces of *f*_H_ such as a bell shape (without abrupt ascending and descending slopes of *f*_H_; figure 1*c*) with much variation in *f*_H_. This variation is caused by momentary bradycardia that occurs each time the bird submerges its head (Stephenson *et al.* 1986). Moreover, Ely *et al.* (1999) observed that antagonistic social interactions between geese (*Anser albifrons*) could rapidly increase heart rate to 400 beats per minute within a few seconds (1–2.5 s). However, since we considered only heart rate events longer than 18 s as a flight, we most probably excluded these types of behaviours.

Our results illustrate that outside of their migration periods, eider ducks fly for a very small proportion of the day. Flight frequency is particularly high around sunrise, decreases during the day and drops markedly during the night (figure 2). This pattern is matched, as hypothesized, by diving frequency. Common eiders are benthic predators, feeding mostly on blue mussels (*Mytilus edulis*), a sessile prey (Guillemette *et al.* 1992). As they feed little during the night (figure 2), they are probably drifting on the sea surface away from the food patches. As the starvation level increases throughout the night, hunger is at its highest level in the morning forcing the birds to forage again. Since coastal currents are tightly related to winds and tide levels, drifting away from the food patches during resting bouts (Guillemette *et al.* 1992) may explain the match between flight and diving frequency observed during day and night.

A detailed analysis of this relationship is beyond the scope of this preliminary report. For instance, in another contribution, we test the hypothesis that dive frequency between flights follows a lognormal distribution with a high frequency of dives right after a flight followed by much lower foraging activity before the next flight. Such a distribution of dive frequency is expected when flight costs are excessive (Pelletier 2006), which can be exacerbated by the accumulation of prey in the digestive system (Guillemette 1994). Nevertheless, we suggest here that flight behaviour of common eiders is an integral component of their foraging strategy.

## Figures and Tables

**Figure 1:**
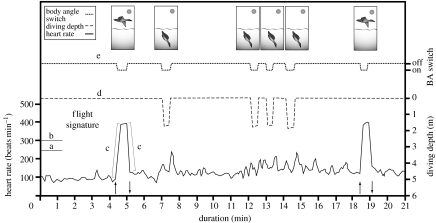
Traces of heart rate (*f*_H_), diving depth and body angle from a female common eider during dives and forward flapping flight. The flight signature is determined by the following parameters: (a) *f*_H_ threshold equal to or above 250 beats per minute, (b) *f*_H_ plateau threshold equal to or above 300 beats per minute, (c) *f*_H_ ascending and descending slopes equal to or above 10 beats per minute per second (absolute values), (d) standard deviation of diving depth up to 0.1 m (§2), and (e) body angle switch (BA switch) turned on. The upward and downward pointing arrows indicate the point of take-off and landing, respectively.

**Figure 2:**
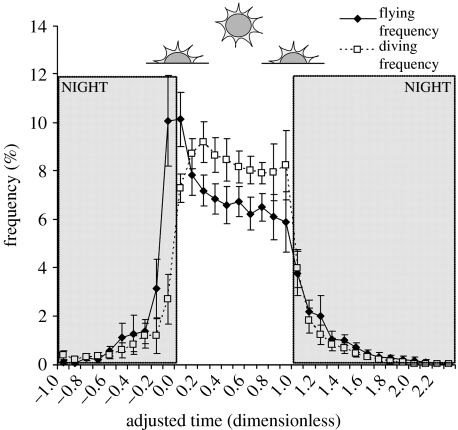
Mean relative flying and diving frequencies (means±95% CI, %) recorded in 13 common eiders according to adjusted time of the day (where ‘0’ is sunrise and ‘1’ is sunset). These behaviours are performed outside the migration and moult periods from May to December 2003. See text for further details.

## Supplementary Material

Heart rate of common eiders engaged in 11 different behaviours Heart rate (means±95% CI, beats per minute) of common eiders engaged in 11 different behaviours (332 recordings, 4 to 86 recordings per behaviour). Asterisks indicate the statistical difference between flight and other activities (ANOVA—Tukey; ^*^: *p*<0.02, ^***^: *p*<0.001 and n.s.: *p*>0.05). Values in parentheses are number of individuals observed per behaviour
