# Supplementary material for: It is time to move: linking flight and foraging behaviour in a diving bird
Source: Biol Lett. 2007 May 15;3(4):357–9. doi: 10.1098/rsbl.2007.0088 (PMC2111053; doi:10.1098/rsbl.2007.0088)
Supplement: Supplementary file 1 [file rsbl20070088s02.doc]

**ELECTRONIC APPENDIX**

This is the Electronic Appendix to the article

# It’s time to move: linking flight and foraging behaviour in a diving bird

by

David Pelletier, Magella Guillemette, Jean-Marc Grandbois and Patrick J. Butler

*Biol. Lett.* (doi:rsbl.2007.0088)

Electronic appendices are refereed with the text; however, no attempt is made

to impose a uniform editorial style on the electronic appendices.

**Supplementary Online Material**

We compared *f*_H_ recorded during flight to *f*_H_ recorded during different activities by conducting focal observations (only one animal at a time) on 14 experimental females during the post-hatching period in 2003 and 2004 (total of 118 h of observation). Behaviours were assigned to one of 11 categories: (1) aggression (between conspecifics, instigator or victim, instigator has head fully extended towards the victim, hitting or biting with bill, on water or land), (2) alarm (head up, neck fully extended, back horizontal to ground (or water) or chest higher than tail), (3) bathing (swimming on water with wing flapping and head submersion), (4) dabbling (head completely into water with body vertical to water or not), (5) diving (bird totally submerged), (6) flying (bird in the air), (7) pausing between dives (floating on water for several seconds between two successive dives throughout a foraging [diving] sequence), (8) resting (neck coiled on back or head and neck on back with bill pointing towards tail), (9) standing or preening (head up and not moving or manipulating feathers with bill, on water or land), (10) swimming (slow or fast movement on water) and (11) walking (slow or fast movement on land).

Overall, there was significant variation in *f*_H_ among behaviours (*F*_10,59_ = 13.58, *p* < 0.001). Mean *f*_H_ during flight (374 ± 17 beats min^-1^), bathing (293 ± 50 beats min^-1^) and between two successive dives (332 ± 50 beats min^-1^) were not significantly different from each other. Nevertheless, the non-significant lower *f*_H_ of bathing was probably due to the small sample size (*N* = 3) and pausing between dives could be discriminated from flying behaviour with diving depth recordings.

Heart rate (means ± 95% CI, beats min^-1^) of common eiders engaged in 11 different behaviours (332 recordings, 4 to 86 recordings per behaviour). Asterisks indicate the statistical difference between flight and other activities (ANOVA - Tukey; * : p < 0.02, *** : p < 0.001 and NS : p > 0.05). Values in parentheses are number of individuals observed per behaviour***.***
